# NT3 treatment alters spinal cord injury-induced changes in the gray matter volume of rhesus monkey cortex

**DOI:** 10.1038/s41598-022-09981-5

**Published:** 2022-04-08

**Authors:** Shu-Sheng Bao, Can Zhao, Hao-Wei Chen, Ting Feng, Xiao-Jun Guo, Meng Xu, Jia-Sheng Rao

**Affiliations:** 1 Beijing Key Laboratory for Biomaterials and Neural Regeneration, Beijing Advanced Innovation Center for Biomedical Engineering, School of Biological Science and Medical Engineering, Beihang University, Beijing, 100083 China; 2 Institute of Rehabilitation Engineering, China Rehabilitation Science Institute, Beijing, 100068 China; 3 School of Rehabilitation, Capital Medical University, Beijing, 100068 China; 4 Department of Orthopedics, The First Medical Center of PLA General Hospital, Beijing, 100853 China

**Keywords:** Diseases of the nervous system, Regeneration and repair in the nervous system

## Abstract

Spinal cord injury (SCI) may cause structural alterations in brain due to pathophysiological processes, but the effects of SCI treatment on brain have rarely been reported. Here, voxel-based morphometry is employed to investigate the effects of SCI and neurotrophin-3 (NT3) coupled chitosan-induced regeneration on brain and spinal cord structures in rhesus monkeys. Possible association between brain and spinal cord structural alterations is explored. The pain sensitivity and stepping ability of animals are collected to evaluate sensorimotor functional alterations. Compared with SCI, the unique effects of NT3 treatment on brain structure appear in extensive regions which involved in motor control and neuropathic pain, such as right visual cortex, superior parietal lobule, left superior frontal gyrus (SFG), middle frontal gyrus, inferior frontal gyrus, insula, secondary somatosensory cortex, anterior cingulate cortex, and bilateral caudate nucleus. Particularly, the structure of insula is significantly correlated with the pain sensitivity. Regenerative treatment also shows a protective effect on spinal cord structure. The associations between brain and spinal cord structural alterations are observed in right primary somatosensory cortex, SFG, and other regions. These results help further elucidate secondary effects on brain of SCI and provide a basis for evaluating the effects of NT3 treatment on brain structure.

## Introduction

Traumatic spinal cord injury (SCI) refers to the trauma-induced damage to the spinal cord structure and function, which may lead to impairment of sensory and motor functions below the injury level^1^. After primary SCI (the mechanical trauma at the “epicenter” of the damage), resultant cascades of cellular and molecular events known as secondary SCI^2^, such as neuronal death^3^, demyelination, and nerve fiber degeneration^4^, may occur at and beyond the injury level^5^. These usually cause further effects on neural structures beyond the injury level, such as the brain, resulting in sensorimotor disorders, neuropathic pain (NP), and other complications^6^. The accurate observation of such structural and functional alterations facilitates the assessment of the injury/recovery status of clinical patients.

MRI has been widely used for non-invasive inspection of the central nervous system and histological validations have demonstrated its reliability^7–11^. Where voxel-based morphometry (VBM)^12^ is currently one of the most popular methods used for the quantitative investigation of brain meso-structure. It transforms the high-resolution 3D MRI images into a unified standard space to conduct voxel-wise quantitative analysis, enabling the accurate detection of brain structural alterations non-invasively. Many researchers have used it to explore brain structural alterations post-SCI^13–15^. In 2004, Crawley et al.^16^ explored the gray matter volume (GMV) alterations in the primary motor cortex (M1) of cervical cord injured patients by using VBM. The GMV of M1 in patients was reduced by only approximately 3.8% compared with that of the controls, without statistical significance. They therefore suggested that functional remodeling rather than anatomical alterations was more likely to occur within M1 post-SCI. Freund et al.^17^ first used VBM to detect longitudinal neurodegenerative processes above the injury level after acute SCI. They conducted 1 year follow-up to explore gray and white matter atrophy in the corticospinal tract (CST) and sensorimotor cortex of the patients and the controls. At the level of the internal capsule and the right cerebral peduncle, the volume loss of CST white matter and left M1 gray matter was faster in the patients than in the controls. This longitudinal study explored the spatiotemporal pattern of the neurodegenerative process post-SCI and identified widespread upstream atrophy and meso-structural alterations that occurred within corticospinal axons and sensorimotor cortical regions of the patients within the first few months after the injury. Besides the intensive exploration of classical sensorimotor pathways^18^, researchers focusing on VBM post-SCI have recently been exploring common post-SCI complications, such as NP^19,20^, psychological, cognitive impairment^13,15^, and their related brain regions. Meanwhile, several studies have explored the possible effects of factors, such as the extent, level, and duration of SCI, on the cortical alterations in patients to address the discrepancy among outcomes reported in different publications^14,21^; these studies further demonstrated the complexity of structural and functional changes within the brain post-SCI.

Although the VBM study post-SCI has been extensive, some problems still exist in the current research. First, most of the relevant studies were cross-sectional^22–24^, and longitudinal studies^17,25^ were relatively few. The brain alterations post-SCI were a progressive process, and the cross-sectional studies targeting a single time point provided limited guidance for the exploration of the changing process. Second, many studies only considered the effects of injury-induced spontaneous plasticity on brain structure. Some of the publications involving rehabilitation treatment only focused on the improvement of motor function in patients^26,27^. Few studies focused on the effects of tissue regeneration in spinal cord on brain cortex. In addition, few investigators have explored the alterations in the brain and spinal cord structure simultaneously post-SCI^17,25,28^. Correspondingly, the potential association between the structural changes of brain and spinal cord is less intensively covered by current research.

The bioactive materials (neurotrophin-3 (NT3) coupled chitosan) previously developed by our group have been confirmed to elicit robust activation of endogenous neural stem cells (NSCs) in the injured spinal cord of non-human primates. The bioactive materials attracted NSCs to migrate into the lesion area through slow release of NT3, differentiate into neurons^29^. Enhanced angiogenesis and reduced inflammatory responses also provided an excellent microenvironment for neurons^30^. These biological effects promoted the form of functional neural networks, which interconnected severed ascending and descending axons, resulting in the recovery of sensorimotor function^31^. Therefore, we used this material to build a regenerative treatment model on the basis of rhesus monkeys SCI model in the current study. The meso-structural alterations in the brain of animals caused by SCI and treated with implanted materials were compared through VBM. This study aimed to observe the differences between the effects of spontaneous plasticity (caused by injury) and tissue regeneration (induced by NT3 treatment) on brain structure after SCI and to explore the possible association between the structural alterations in the brain and spinal cord.

## Methods

### Animal model preparation

The experimental animals used in this study were eight adult female rhesus monkeys (mean weight: 5 ± 1 kg, mean age: 5–6 years old). Female monkeys were chosen because they have better coordination and less aggression than males and are easy to care for after injury. Four of the animals were randomly designated as the lesion control (LC) group and underwent SCI. The other four were implanted with repair materials immediately post-SCI and were designated as the NT3 group. Model preparation was performed in a sterile operating room by professional surgeons. Anesthesia of animals was induced by intramuscular injection of ketamine hydrochloride solution (10 mg/kg) before the start of surgery and then maintained by intramuscular injection of xylazine hydrochloride (5 mg/kg). The spinal cord hemi-transection and remove model was used. First, the T7–9 segments of rhesus monkeys (approximately corresponding to the T10–12 segments of the thoracic cord) were located as the injury site. Then, the spinal cord tissue (1 cm long and 2–3 mm wide) was excised 0.5 mm to the right of the posterior central spinal vein to establish the spinal cord hemi-transection and remove model of rhesus monkeys. After topical hemostasis, the repair materials with the corresponding size were implanted into the injury area of the NT3 animals, whereas no intervention was performed on the LC animals. Antibiotics (penicillin, 240 mg/D) were administered for 5 days postoperatively to prevent infection. All rhesus monkeys were individually housed in monkey cages at constant temperature and humidity. We ensured that they received adequate food and fresh fruit. Detailed procedures were presented in the previous publication^31^.

### MRI scanning

All datasets were collected with the Siemens 3.0 T magnetic resonance system (Magnetom Trio Tim, Siemens, Erlangen, Germany). During scanning, a custom-made four-channel primate head birdcage coil was initially used to acquire brain structural images. Then, a human spinal standard array transmitter and receiver coil was used to obtain spinal cord structural images. Considering the stability of the physiological state of the animals, the scan time points were set as follows: healthy period (baseline); and 1, 2, 3, 6, and 12 months post-SCI.

To obtain brain images, animals were intramuscularly injected with ketamine hydrochloride solution (10 mg/kg) prior to the scanning to induce anesthesia. An intramuscular injection of atropine sulfate (0.05 mg/kg) was administered to reduce salivary secretion. Then, venipuncture and continuous administration of a mixed saline solution of propofol (0.25 mg/kg/min) and ketamine (0.03 mg/kg/min) were conducted to maintain anesthesia^31^. Anesthetized rhesus monkeys were placed inside the scanning cavity in the “Sphinx” posture. The head of the animal was placed and fixed in the birdcage coil, and the breathing patency was guaranteed. The respiration and heart rate of animals were monitored throughout the scanning period to ensure that the respiratory rate was maintained at above 20 beats/min, and the heart rate was maintained at above 70 beats/min^32^. High resolution sagittal T1 weighted images were acquired using the 3D magnetization-prepared rapid acquisition gradient echo (MPRAGE) sequence with the following imaging parameters: repetition time (TR) = 1850 ms; echo time (TE) = 4.85 ms; flip angle = 8°; inversion time (TI) = 800 ms; matrix = 256 × 256; field of view (FOV) = 120 × 120 mm, slices = 160; and resolution = 0.47 × 0.47 × 0.5 mm^3^. Parts of the raw brain images were shown in Fig. S1 (Supporting Information).

To obtain the spinal cord images, anesthetized rhesus monkeys were placed inside the scanning cavity in the supine posture. Proton-density weighted sequence was used to acquire axial spinal cord structural images with the following parameters: TR = 3050 ms; TE = 11 ms; flip angle = 15°; matrix = 320 × 320; FOV = 196 × 196 mm, and resolution = 0.6 × 0.6 × 2.0 mm^3^. Scanning center was located at the surgical position of the spinal cord, and 27 consecutive slices of axial images covering the SCI region were collected. A saturation belt was set at the thoracoabdominal region to suppress the susceptibility artifacts caused by the air tissue interface. Brain and spinal cord scanning took approximately 20 min in total.

### Function evaluation

The alterations of sensorimotor function were evaluated by collecting the withdrawal thermal thresholds (WTT) and step height of LC and NT3 animals at baseline, 1 month and 12 months post-SCI.

#### WTT

The WTT of LC and NT3 animals were collected by stimulating the left (contralateral to the injury) hindlimbs with the DS2-21612–105 semiconductor laser (BWT Beijing Ltd, Beijing, CN). The infrared laser wavelength was 810 nm, and the distance from the fiber port of the ranging laser to the skin was 1 cm, with a spot diameter of about 5 mm. The current value was initially set to 6.5 A and gradually increased with a gradient of 0.75 A, with the maximum current value limited to 11 A. To avoid scald, the stimulation time for each gradient was not allowed to exceed 30 s. When a significant withdrawal reflex appeared in 30 s, the machine stopped delivering the stimulation and the current values were recorded. Otherwise, the machine terminated the trial and restarted it from the next gradient after a 5-min inter-trial interval.

#### Step height

The stepping of the right (ipsilateral to the injury) hindlimbs was collected using the Vicon system (Vicon 8, Oxford Metrics Limited Company, Yamton, UK). A reflective marker was attached to the second metatarsophalangeal joint and its spatial position was recorded in real time by multiple cameras (recording frequency: 100 Hz). The animals were allowed to walk on a treadmill bipedally (with upper body restrained, speed: 0.5 m/s) and successive stepping (> 5 steps) data were collected for subsequent analysis.

### Data processing

Statistical parametric mapping (SPM) version 12 (University College London, London, UK) was used to preprocess MRI images of the rhesus monkey brain. The VBM preprocessing pipeline for inter-group comparison of brain structures at each time point included the following steps. First, all T1 weighted structural images were registered with the INIA19 template^33^ using the rigid body model for them to have the same standard space. The registered images were subjected to unified segmentation^34^ using the tissue probability maps provided by the template to obtain separate gray matter segments. Then, Diffeomorphic Anatomical Registration Through Exponentiated Lie algebra (DARTEL)^35^ was used to average and warp the gray matter images six times to generate a study-specific template (SST). The SST was transformed into the Montreal Neurological Institute space by affine registration. The gray matter images were normalized to the SST by using the flow field generated by DARTEL and were modulated by Jacobian determinant to preserve the actual tissue volume. Finally, the normalized images were smoothed using Gaussian kernels of 2 mm full width at half maximum (FWHM).

The VBM preprocessing pipeline used to explore the spatiotemporal pattern of longitudinal brain structural alterations post-SCI was similar to that used in the abovementioned procedure, but some differences existed. First, following the initial registration with the template, the anatomical images of each animal at six time points were longitudinally registered to the generated mid-point average image. Considering the multiple time points in the research, a velocity field divergence (dv) map, instead of Jacobian determinant for each time point was generated to represent the rate of volumetric alteration of each time point relative to the mid-point average image; positive values indicated expansion, whereas negative values indicated contraction. Then, the mid-point average images were segmented, and the SST was created. The dv maps were then multiplied by the gray matter segment of mid-point average images to obtain the GMV alteration images, which could indicate the longitudinal alteration tendencies of GMV with time and were proved to be more sensitive in small sample size studies^36^. Afterward, the GMV alteration maps were normalized, modulated, and smoothed (2 mm FWHM) as described above. We explored brain regions with different longitudinal alteration tendencies of GMV in the LC and NT3 animals for over 1 year and extracted the overall GMV of these brain regions.

Spatially adaptive non-local means filter was used to denoise while maintaining the detail features of spinal cord images to the maximum degree^37^. To measure the cross-sectional spinal cord area (SCA), the regions of interest (ROI) were selected at the epicenter, 2 cm rostral, and 2 cm caudal of the injury area. Considering the irregular structure of spinal cord in the injury area, automatic extraction of spinal cord was relatively difficult. Therefore, the SCA was delineated and calculated by manual outlining in Photoshop CC 2018 (Adobe Systems Inc., California, USA). All manual outlining processes were completed by a medical imaging professional who was not involved in this experiment. After SCA measurement, the cross-sectional left–right width (LRW) and anterior–posterior width (APW) of the spinal cord were further extracted for subsequent analysis.

Gait datasets were processed and computed using custom software based on Matlab (MathWorks, Natick, MA, USA). Gait cycle were first automatically divided^38^. The step height for each gait cycle was then calculated, which was used for subsequent evaluation of the motor functional recovery in both groups.

### Statistics

SPM12 and Data Processing & Analysis for Brain Imaging (DPABI)^39^ were used for the statistical analysis of brain quantitative parametric maps. Two sample t-test implanted in DAPBI was used to compare the GMV and its alteration between LC and NT3 groups at each time point. Total intracranial volume was set as the covariate to exclude the interference of heterogeneity in brain size among individuals. Gaussian random field (GRF) theory was used for multiple comparison correction, with voxel-level set to p < 0.005 and cluster-level set to p < 0.05.

To analyze the longitudinal GMV alteration tendencies in LC and NT3 animals, we adopted the two-step analysis procedure commonly used in fMRI and longitudinal image analysis^25^. In the first step, we evaluated the images of each animal at all time points using regression analysis implanted in SPM12 to establish the quadratic trajectory model of GMV and time since injury (t): y(t) = β_0_ + β_1_ t + β_2_ t^2^. This process would generate the parametric maps of β_1_ and β_2_ for each animal, where β_1_ represented the linear effect of volume change with time, i.e., the expansion or contraction of volume. β_2_ represented the quadratic effect of volume change with time, i.e., acceleration or deceleration of expansion or contraction. In the second step, parametric maps of β_1_ and β_2_ were input into the one sample t-test in DPABI to detect the brain regions with significant volume alterations over time in LC and NT3 animals. In addition, two sample t-test was used to compare the difference between the two groups in terms of GMV alteration tendency over time. The settings of the covariates and multiple comparison correction were the same as those described in the previous paragraph.

To analyze the association between structural alterations in the brain and spinal cord, correlation analysis in DPABI was conducted to detect the brain regions where significant correlations existed between the GMV changes relative to baseline and the SCA, LRW, and APW alterations relative to baseline. Data from all time points were included in the analysis. GRF theory was used for multiple comparison correction. The voxel-level was set to p < 0.001, and the cluster-level was set to p < 0.05.

SPSS 20 (SPSS Inc., Chicago, IL) software was used for the quantitative analysis of the extracted overall GMV of brain regions, spinal cord structural parameters, WTT and step height. Regression analysis was used to test the linear or quadratic alterations of GMV, SCA, LRW, and APW with time. The linear effect represented the progressive change in the brain or spinal cord structure. The quadratic effect represented the change in the alteration rate. Chow breakpoint test was used to compare the differences in longitudinal spinal cord structural and WTT alteration tendency over time between LC and NT3 groups. This step was conducted through an in-house function in SPSS. To avoid individual heterogeneity, the spinal cord structural parameters and WTT values were divided by the corresponding individual baseline values to obtain the comparable normalized results. One sample Kolmogorov Smirnov (K-S) test was initially used to test the data distribution normality. Then, the variability of brain and spinal cord structural alteration rate between two groups and the step height at 1 month and 12 months in each group were compared (two sample t-test, normal; two sample K-S Z test, non-normal). The WTT values between different timepoints were also compared (paired t-test, normal; Wilcoxon signed rank test, non-normal; both with Bonferroni correction). To explore whether the spinal cord atrophy in NT3 group had reversed that in LC group to some degree, the parameters at 12 months of LC and NT3 groups and baseline were compared (one-way analysis of variance (ANOVA) with Bonferroni correction). The Spearman correlation analysis was used to verify the correlation among the GMV changes relative to baseline and spinal cord structural alterations relative to baseline. The association between the GMV and WTT was also analyzed by Spearman correlation analysis. The significance threshold was set to p < 0.05. The results were given as mean ± s.e.m.

### Ethics approval and consent to participate

All experimental procedures were approved by the Biological and Medical Ethics Committee of Beihang University (BM20180046). All experiments were performed in accordance with relevant named guidelines and regulations. The authors complied with the ARRIVE guidelines.

## Results

### GMV differences between LC and NT3 treatment animals

**Figure 1:**
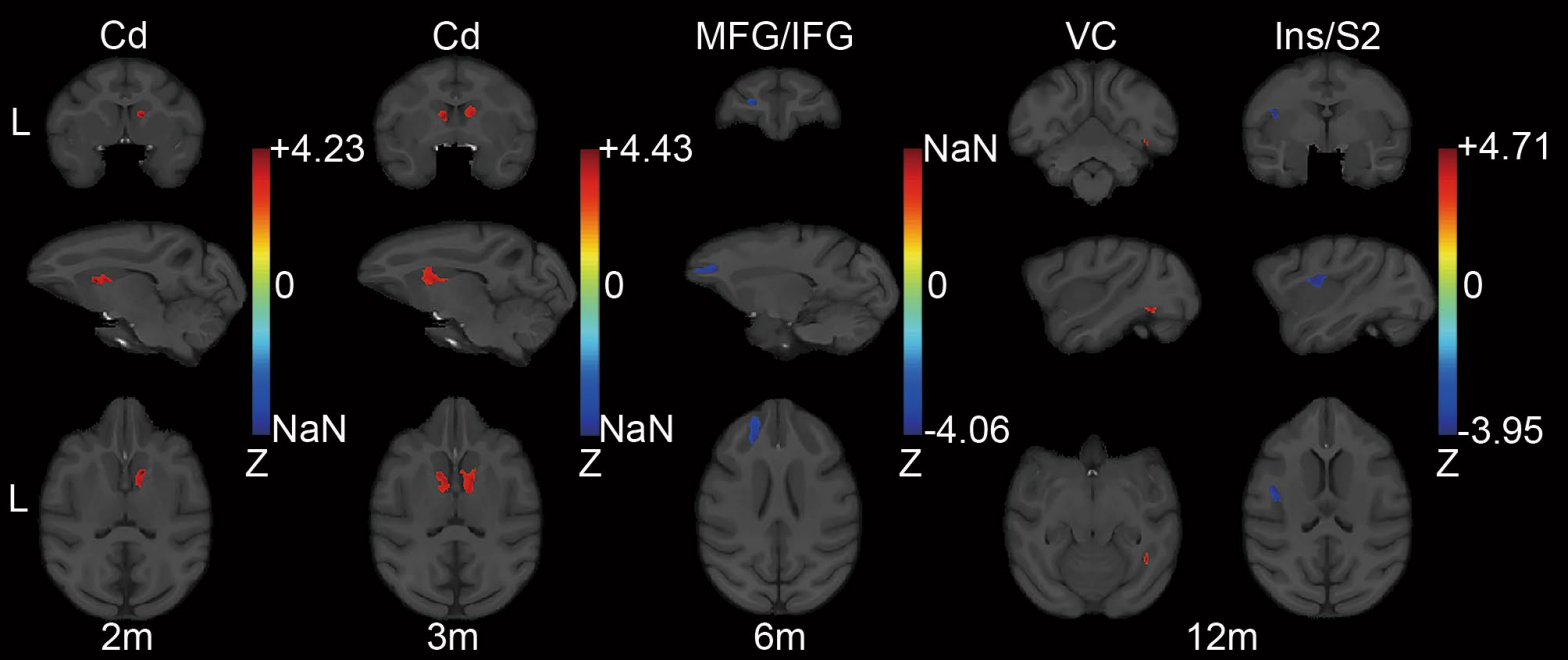
GMV differences between LC and NT3 animals overlaid on the T1 template image. Only clusters with significant differences were displayed (two-sample t-test with GRF correction). The voxel level was set to p < 0.005, and the cluster level was set to p < 0.05. The brain regions where the significant clusters were located were presented above the images; m represented the month; a positive value in the Z-bar indicated that LC > NT3; L indicated the left side; *Cd* caudate nucleus, *MFG* middle frontal gyrus, *IFG* inferior frontal gyrus, *VC* visual cortex, *Ins* insula, *S2* secondary somatosensory cortex.

There was no significant difference in GMV between the two groups of NT3 and LC at the baseline (Fig. S2, Supporting Information). After SCI, significant differences were found between the GMV of animals in the LC and NT3 groups across a wide range of brain regions, including the cerebral cortex and basal ganglia. The GMV of the LC in the right caudate nucleus (Cd) was significantly greater than that of the NT3 group at 2 months after injury. This tendency also appeared in the left Cd at 3 months after injury. The differences in Cd were no longer significant at 6 months after injury. Meanwhile, the regions where the GMV of the LC was smaller than that of the NT3 animals appeared for the first time in left middle frontal gyrus (MFG) and inferior frontal gyrus (IFG). At 12 months after injury, the GMV of the LC on the right visual cortex (VC) was significantly greater than that of the NT3 group, whereas the GMV of the LC on the left insula (Ins) and secondary somatosensory cortex (S2) was significantly lower than that of the NT3 animals (Fig. 1). Table 1 presents the details of significant clusters.

**Table 1:** GMV differences between the LC and NT3 animals.

	Months	Z score	Cluster extent(voxels)	x (mm)	y (mm)	z (mm)
Cd_R	2	4.23	321	6	24.5	22
Cd_L	3	4.43	340	− 3	21.5	22
Cd_R	3	3.78	716	6	22	22.5
MFG/IFG_L	6	− 4.06	353	− 16.5	29.5	30.5
VC_R	12	4.71	107	18.5	− 5	13.5
Ins/S2_L	12	− 3.95	293	− 17	19	24

*L* left, *R* right, *Cd* caudate nucleus, *MFG* middle frontal gyrus, *IFG* inferior frontal gyrus, *VC* visual cortex, *Ins* insula, *S2* secondary somatosensory cortex.

### Longitudinal alterations in brain structure

**Figure 2:**
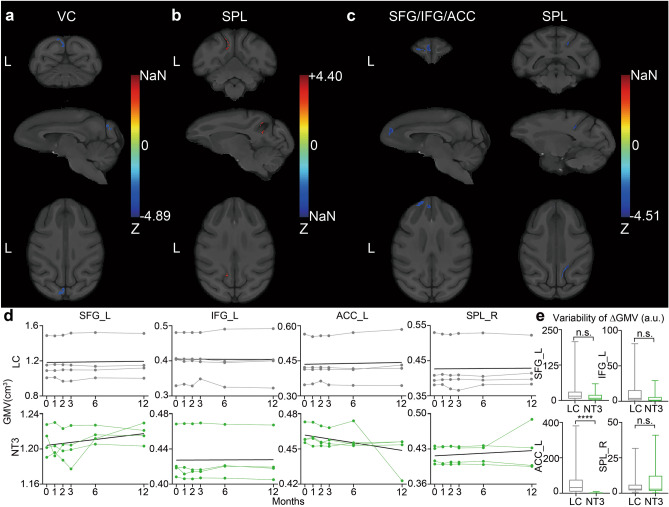
Longitudinal GMV alterations of LC and NT3 animals over 1 year after injury. (**a**) The brain regions in which the GMV of the LC significantly decreased over time within 1 year. (**b**) The brain regions in which the GMV of the NT3 group significantly increased over time within 1 year. (**c**) The brain regions in which the GMV alteration showed significant differences between the two groups. Only clusters with significant differences were displayed. One-sample t test was used for intra-group modeling, and two-sample t test was used for inter-group comparison, with GRF correction. The voxel level was set to p < 0.005, and the cluster level was set to p < 0.05. The brain regions where the significant clusters were located are presented above the images. A positive value in the Z-bar indicated that LC > NT3; L indicated the left side. d) The results of longitudinal alterations in GMV in the significantly different brain regions. e) The results of inter-group comparisons on the variability of GMV alteration rate in the significantly different brain regions (two-sample K-S Z test). ****p < 0.0001; *VC* visual cortex, *SPL* superior parietal lobule, *SFG* superior frontal gyrus, *IFG* inferior frontal gyrus, *ACC* anterior cingulate cortex, *LC* lesion control, *NT3* neurotrophin-3, *GMV* gray matter volume.

Within 1 year after injury, a significant linear effect of regional GMV alteration over time was found on LC and NT3 animals, i.e., the rate of regional GMV alteration in each group was stable. No significant quadratic changes were found. The GMV of left VC decreased significantly over time in LC (Fig. 2a), whereas that of left superior parietal lobule (SPL) increased significantly over time in NT3 group (Fig. 2b). The GMV alterations of the LC in the superior frontal gyrus (SFG), IFG, anterior cingulate gyrus (ACC), and right SPL were significantly lower than those of the NT3 animals (Fig. 2c). Specifically, the GMV of these clusters decreased in the LC, while it increased in the NT3 group. The results of longitudinal alterations in GMV in the significantly different brain regions were shown in Fig. 2d. The comparison of the GMV alteration rate variability in these brain regions showed that in the left ACC, the alteration rate of the LC was much more varied than that of the NT3 group (p = 0.6 × 10^–5^). No significant differences were found in the variability of left SFG, IFG, or right SPL between the two groups (Fig. 2e). Table 2 presents the details of significant clusters.

**Table 2:** Longitudinal brain structural alterations in the LC and NT3 animals.

	Z score	Cluster extent (voxels)	x (mm)	y (mm)	z (mm)
**LC**					
VC_L	− 4.89	528	− 2	− 14.5	29
**NT3**					
SPL_L	4.40	175	− 7	1	29.5
**LC vs. NT3**					
SFG/IFG/ACC_L	− 4.43	183	− 2	43	26.5
SPL_R	− 4.51	158	8.5	1.5	30.5

*LC* lesion control, *NT3* neurotrophin-3, *L* left, *R* right, *VC* visual cortex, *SPL* superior parietal lobule, *SFG* superior frontal gyrus, *IFG* inferior frontal gyrus, *ACC* anterior cingulate cortex.

### Longitudinal alterations in spinal cord structure

**Figure 3:**
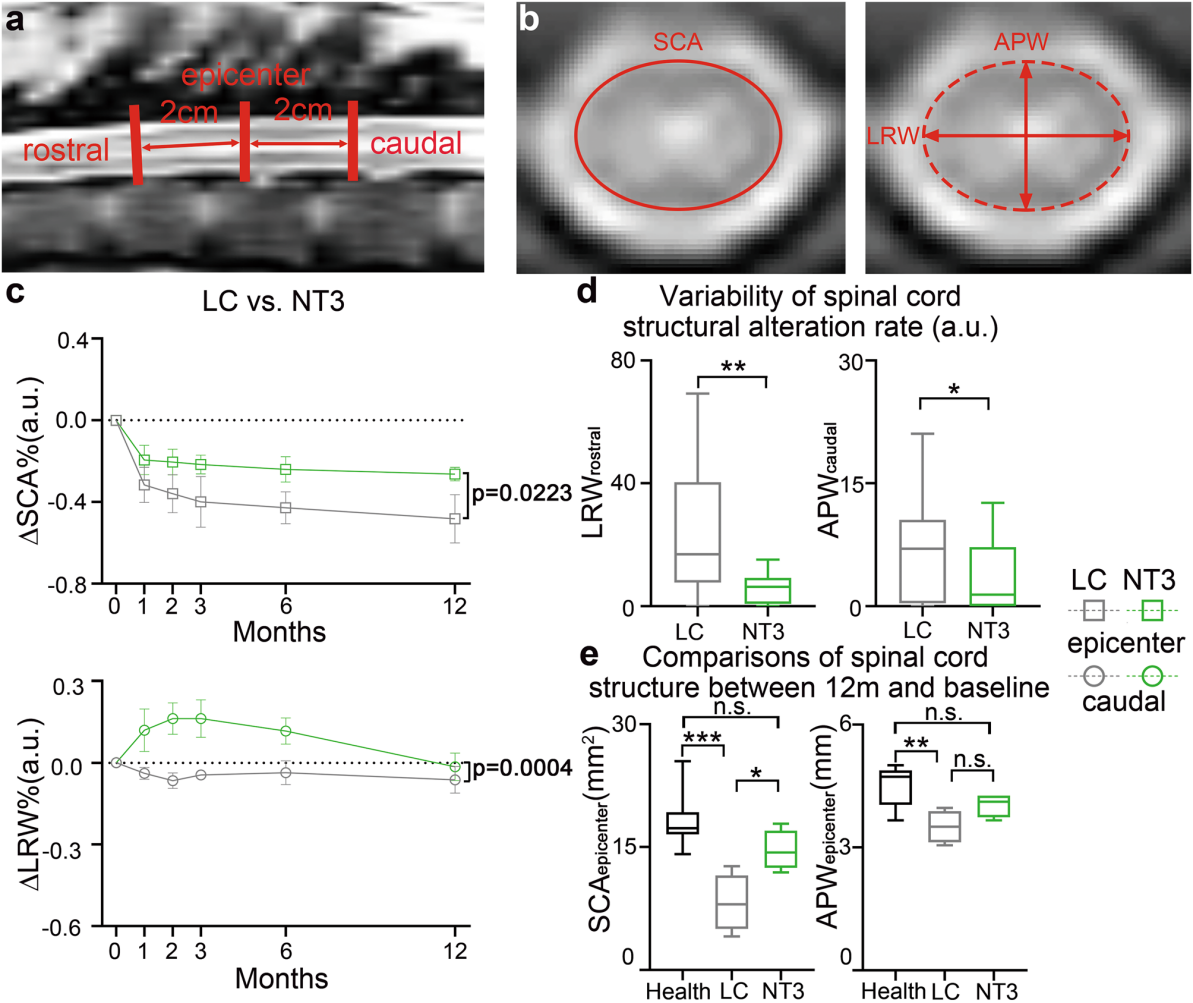
Differences in longitudinal SC structural alterations between LC and NT3 animals within 1 year post-SCI. (**a**) A schematic diagram of measurement of spinal cord structural parameters at the epicenter, 2 cm at the rostral, and 2 cm at the caudal of injury area. (**b**) The schematic diagram of measurement of SCA, LRW, and APW. (**c**) Differences in the longitudinal spinal cord structural alteration tendencies between LC (gray) and NT3 animals (green) (Chow breakpoint test). To avoid individual heterogeneity, the spinal cord structural parameters were divided by the corresponding individual baseline values to obtain the comparable normalized results. (**d**) Inter-group differences in the variability of spinal cord structural alterations (two-sample K-S Z test). (**e**) Differences in the spinal cord structural parameters between 12 m and baseline (one-way ANOVA with Bonferroni correction). Only parameters with significant difference were presented. *p < 0.05; **p < 0.01; ***p < 0.001; m represented the month; *LC* lesion control, *NT3* neurotrophin-3, *SCA* spinal cord area, *LRW* left–right width, *APW* anterior–posterior width.

The measurement of spinal cord structural parameters was shown in Fig. 3a and b. After SCI, the morphology of spinal cord at the epicenter of injury area has been damaged (Fig. S3a, Supporting Information). To avoid individual heterogeneity, the spinal cord structural parameters were divided by the corresponding individual baseline values to obtain the comparable normalized results. Within 1 year after injury, the spinal cord structural parameters of LC and NT3 animals showed obvious quadratic effects with time, including SCA (p = 0.0046) at the epicenter of injury area in LC, SCA (p = 0.0146) and APW (p = 0.0382) at the epicenter of injury area in NT3 group, as well as LRW (p = 0.0068) at the caudal of injury area in NT3 animals. For SCA at the epicenter of injury area of both groups, significant linear components were also detected (LC: p = 0.0107; NT3: p = 0.0389). (Fig. S3b, Supporting Information). This indicated that the rate of spinal cord structural alteration was not constant, but showed a significant acceleration or deceleration. A significant difference was found in the longitudinal spinal cord structural alteration tendencies between the two groups, which was manifested in SCA (p = 0.0223) at the epicenter of injury area and LRW (p = 0.0004) at the caudal of injury area, while no significant difference was found in APW (Fig. 3c). Inter-group comparison of the variability of the spinal cord structural alteration rate revealed that in the LRW (p = 0.0047) at the rostral and in the APW (p = 0.0348) at the caudal of injury area, the alteration rate of the LC was much more varied than that of the NT3 group (Fig. 3d). In the SCA (p = 0.0006) and APW (p = 0.0069) at the epicenter of injury area, significant differences were found between LC group at 12 m and baseline, while NT3 group showed no difference with baseline. Significance was also found in the epicenter SCA (p = 0.0421) between LC and NT3 animals at 12 months (Fig. 3e).

### Correlation between structural alterations in brain and spinal cord

**Figure 4:**
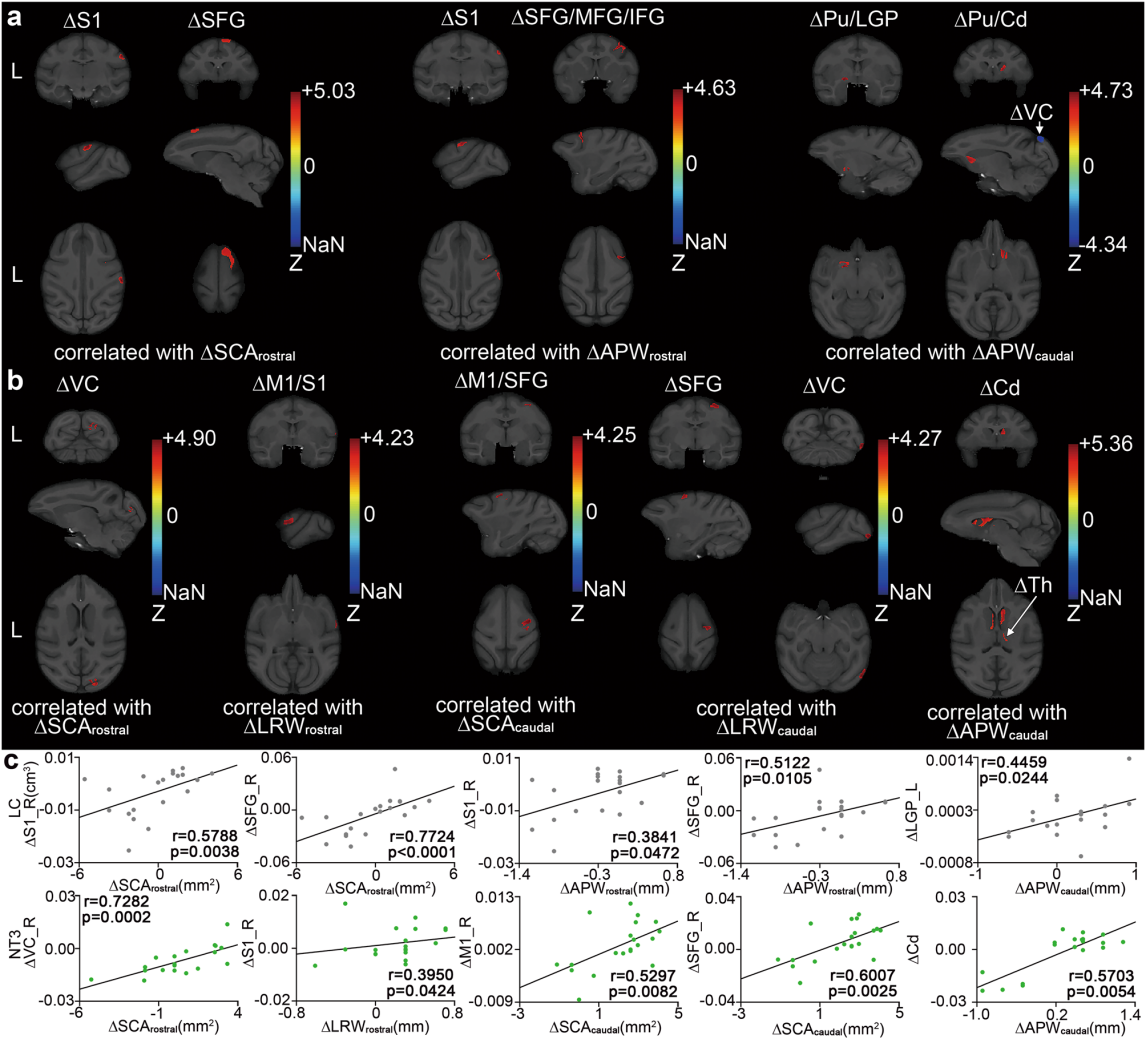
Correlations between brain and spinal cord structural alterations in LC and NT3 animals. (**a**) The brain regions of the LC animals where GMV alterations were significantly correlated with spinal cord structural alterations. (**b**) The brain regions of NT3 animals where GMV alterations were significantly correlated with spinal cord structural alterations. Only clusters with significant correlation were displayed. Correlation analysis with GRF correction; the voxel level was set to p < 0.001, and the cluster level was set to p < 0.05. The brain regions in which the significant clusters were located are presented above the images or indicated by the arrow. The correlated spinal cord structural parameters were presented below the images. A positive value in the Z-bar indicated that LC > NT3. L indicated the left side. (**c**) The results of the correlation analysis between the overall GMV alterations in the above brain regions and the spinal cord structural alterations. Only brain regions with significant correlation were presented (Spearman correlation analysis). *LC* lesion control, *NT3* neurotrophin-3, *SCA* spinal cord area, *LRW* left–right width, *APW* anterior–posterior width, *S1* primary somatosensory cortex, *SFG* superior frontal gyrus, *MFG* middle frontal gyrus, *IFG* inferior frontal gyrus, *Pu* putamen, *LGP* lateral globus pallidus, *Cd* caudate nucleus, *VC* visual cortex, *M1* primary motor cortex, *Th* thalamus.

In the LC and NT3 animals, the distribution of brain regions where a significant correlation existed between GMV alterations and spinal cord structural alterations was relatively wide. For the LC, these regions included the following: right S1, SFG (positive correlation with alteration in rostral SCA); right S1, SFG, MFG, and IFG (positive correlation with alteration in rostral APW); and left lateral globus pallidus (LGP), right Cd, bilateral putamen (Pu) (positive correlation with alteration in caudal APW), and right VC (negative correlation with alteration in caudal APW) (Fig. 4a). For the NT3 animals, these regions included the following: right VC (positive correlation with alteration in caudal SCA); right primary motor cortex (M1) and S1 (positive correlation with alteration in rostral LRW); right M1 and SFG (positive correlation with alteration in caudal SCA); right SFG and VC (positive correlation with alteration in caudal LRW); and bilateral Cd and thalamus (Th) (positive correlation with alteration in caudal APW) (Fig. 4b). Table 3 presents details of significant clusters.

**Table 3:** Correlation between structural alterations in the brain and spinal cord.

	Correlated with	Z score	Cluster extent (voxels)	x (mm)	y (mm)	z (mm)
**LC**						
∆S1_R	∆SCA_rostral_	5.03	243	25	11.5	29
∆SFG_R	∆SCA_rostral_	3.83	562	4	30	39
∆S1_R	∆APW_rostral_	4.33	204	24.5	13	30
∆SFG/MFG/IFG_R	∆APW_rostral_	4.63	291	15	24.5	33.5
∆Pu/LGP_L	∆APW_caudal_	4.73	179	− 8.5	20.5	14
∆Pu/Cd_R	∆APW_caudal_	4.30	348	6.5	30.5	20.5
∆VC_R	∆APW_caudal_	− 4.34	1144	8.5	− 15	35
**NT3**						
∆VC_R	∆SCA_rostral_	4.90	221	6	− 16.5	24.5
∆M1/S1_R	∆LRW_rostral_	4.23	165	27.5	20.5	19.5
∆M1/SFG_R	∆SCA_caudal_	4.25	398	15	18.5	35.5
∆SFG_R	∆LRW_caudal_	4.10	201	12.5	17	37.5
∆VC_R	∆LRW_caudal_	4.27	275	24.5	− 13.5	12.5
∆Cd_L	∆APW_caudal_	4.49	363	− 2	27.5	21.5
∆Cd_R	∆APW_caudal_	5.36	702	3	29.5	23
∆Th	∆APW_caudal_	5.16	105	1	18	17.5

*LC* lesion control, *NT3* neurotrophin-3, *L* left, *R* right, *SCA* spinal cord area, *LRW* left–right width, *APW* anterior–posterior width, *S1* primary somatosensory cortex, *SFG* superior frontal gyrus, *MFG* middle frontal gyrus, *IFG* inferior frontal gyrus, *Pu* putamen, *LGP* lateral globus pallidus, *Cd* caudate nucleus, *VC* visual cortex, *M1* primary motor cortex, *Th* thalamus.

To explore whether the association can be generalized to the overall brain regions where these clusters were located, we extracted the GMV alterations of the overall brain regions, and calculated their correlations with the alterations of spinal cord structural parameters. Some of the abovementioned significant correlations were still retained (Fig. 4c). For the LC, the GMV alterations in right S1 and SFG were positively correlated with the alteration in rostral SCA (r = 0.5788, p = 0.0038; r = 0.7724, p < 0.0001); the GMV alterations in right S1 and SFG were positively correlated with the alteration in rostral APW (r = 0.3841, p = 0.0472; r = 0.6141, p = 0.0020); and the GMV alteration in left LGP was positively correlated with the alteration in caudal APW (r = 0.4459, p = 0.0244). For the NT3 group, the GMV alteration in right VC was positively correlated with the alteration in rostral SCA (r = 0.7282, p = 0.0002); the GMV alteration in right S1 was positively correlated with the alteration in rostral LRW (r = 0.3950, p = 0.0424); the GMV alterations in right M1 and SFG were positively correlated with the alteration in caudal SCA (r = 0.5297, p = 0.0082; r = 0.6007, p = 0.0025); and the GMV alteration in bilateral Cd was positively correlated with the alteration in caudal APW (r = 0.5703, p = 0.0054).

### Verification of sensorimotor functional alterations after SCI

After SCI, the WTT and step height changed in both LC and NT3 groups, and correlation existed between these changes and the GMV alterations in some brain regions.

**Figure 5:**
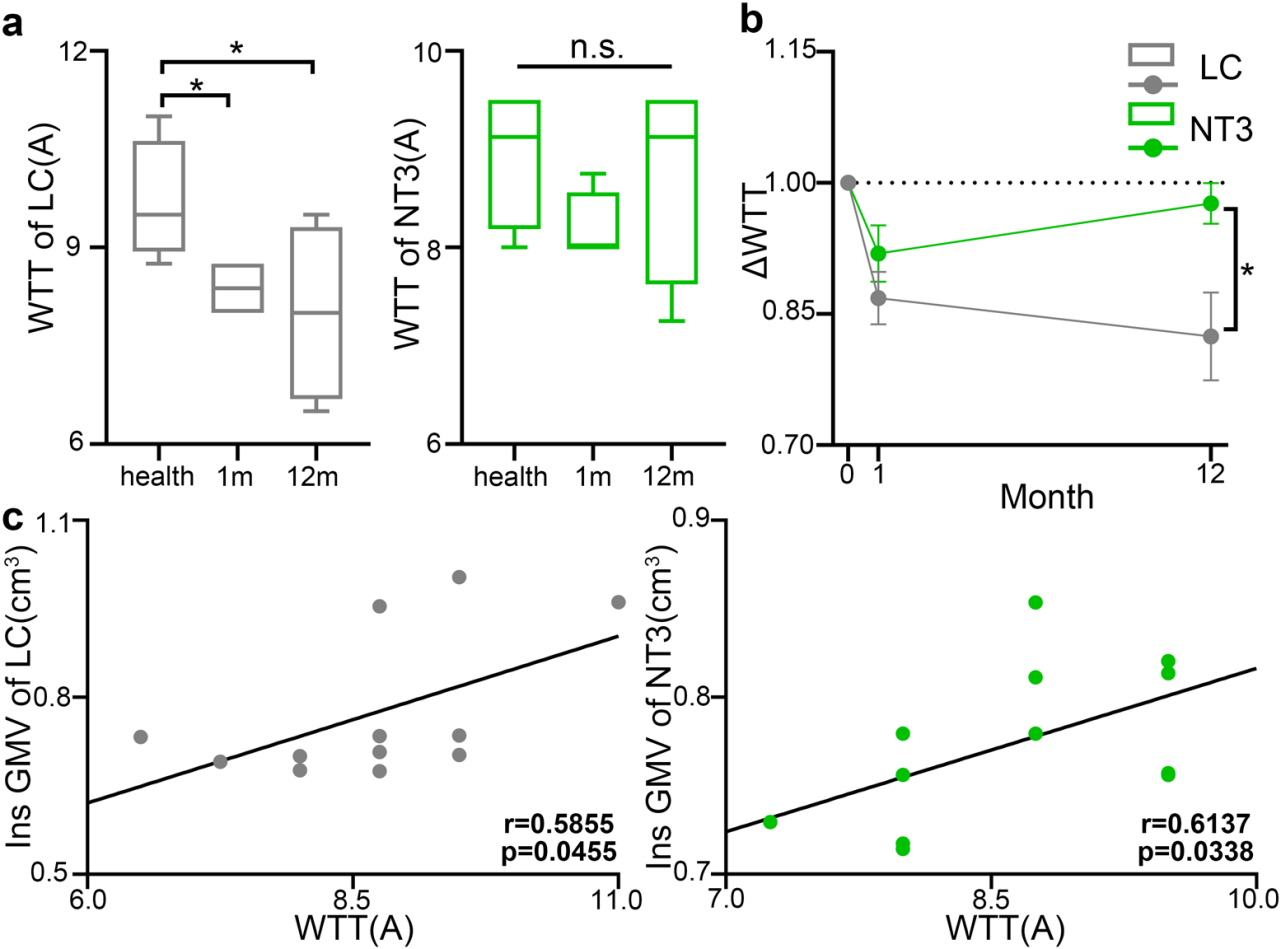
Alteration of WTT in LC and NT3 animals and its correlation with brain GMV. (**a**) Intragroup differences of WTT between baseline, 1 month and 12 months post-SCI (paired t-test with Bonferroni correction). (**b**) Intergroup difference of longitudinal alteration tendency of WTT over time (Chow breakpoint test). (**c**) The significant positive correlation between WTT and the GMV of Ins within both groups (Spearman correlation analysis). *p < 0.05; m represented the month; *LC* lesion control, *NT3* neurotrophin-3, *WTT* withdrawal thermal thresholds, *GMV* gray matter volume, *Ins* insula.

In the LC group, the mean WTT in the healthy period, 1 month and 12 months post-SCI were 9.6875 ± 0.4719 A, 8.3750 ± 0.2165 A, and 8.0000 ± 0.6847 A, respectively. The thresholds at 1 and 12 months after SCI were significantly lower than that in the healthy period (1 month: p = 0.0354; 12 months: p = 0.0374). The mean threshold in the NT3 group at baseline was 8.9375 ± 0.3590 A, with no difference with that in LC animals. At 1 and 12 months post-SCI, the thresholds of NT3 animals were 8.1875 ± 0.1875 A and 8.7500 ± 0.5303 A, respectively, and no significant difference was detected among the three periods (Fig. 5a). After SCI, the longitudinal alteration tendency of WTT showed a significant difference between the LC and NT3 animals (p = 0.0196). The WTT of LC group gradually decreased, while NT3 treatment alleviated the tendency (Fig. 5b). Within both groups, significant positive correlation existed between the overall GMV of Ins and the WTT post-SCI. (LC: r = 0.5855, p = 0.0455; NT3: r = 0.6137, p = 0.0338) (Fig. 5c).

For the LC group, the mean step height was 3.0815 ± 0.4673 mm at 1 month and 8.1625 ± 2.1739 mm at 12 months after SCI, with no significant difference (p = 0.0802). For the NT3 group, the mean step height was 2.6973 ± 0.4668 mm at 1 month and 15.1245 ± 1.8539 mm at 12 months, indicating a significant increase (p = 0.1 × 10^–5^) (Fig. S4).

## Discussion

This study described progressive structural alterations in the brain and spinal cord post-SCI and during NT3 treatment in the spinal cord hemi-transection and remove model of rhesus monkeys. NT3-induced regeneration had unique effects on the structure of the brain and spinal cord compared with SCI. Significant correlations were found between structural alterations in the brain and spinal cord. Given that the clinical imaging of a patient’s injured spinal cord is often constrained by implanted fixtures, the assessment of brain structure may provide additional useful information for monitoring the clinical patient’s spinal cord injury/repairment status.

It should be noted that after SCI, a certain degree of spontaneous plasticity existed in the structure and function of central nervous system, rather than invariable^40–42^. Previous publications have shown that synaptic remodeling, axonal sprouting, and neurogenesis would occur in the spinal cord to some extent after injury^43,44^. Cortex structures would also be remodeled, such as the eliminating of old dendritic spines and the forming of new ones^45^. In this study, the spontaneous plasticity was the main reason for the structural alterations of cerebral cortex in the lesion group. The effects of tissue regeneration were revealed by the changes in the NT3 group, such as the more stable structural alteration rate in spinal cord and brain compared to that in the lesion animals, which may demonstrate the unique changing progress resulting from the implantation of NT3/chitosan.

Though VC and spinal cord were not directly connected, VC still played an important role in the process of performing complex motor tasks, as well as guiding and shaping the movement online. Consequently, there were numerous studies on the functional changes of VC after SCI. For example, Chen et al.^46^ reported decreased functional connectivity (FC) within the medial vision network (mVN) of the brain in patients with incomplete cervical cord injury. Hawasli et al.^47^ found a decrease in FC between VC and M1, as well as VC and sensory parietal cortex post-SCI. These findings were consistent with the results of the present study to some extent. The GMV of the left VC in the LC animals gradually decreased over time, which might reflect the structural atrophy caused by the long-term decline of functional connectivity in VC post-SCI.

Differences were found between the LC and NT3 animals in terms of brain structure at each time point after injury. At 12 months after injury, the GMV of the LC in the right VC was significantly greater than that of the NT3 animals. This phenomenon might be due to the potential effect of VC in pain processing. Preissler et al.^48^ investigated the GMV alterations in the brain of patients with chronic phantom limb pain after amputation, and they found that the GMV in the dorsal and ventral VCs of the patients was significantly larger than that in the healthy control. This phenomenon might reflect a visual compensation mechanism to compensate for the lack of sensorimotor feedback, as SCI patients might need an increased use of visual information to aid in the completion of movement. The same kind of inverted mechanism could be observed in blind subjects, i.e., the loss of visual input enhanced the development of other sensory abilities. In this experiment, the right hemi-transection of the spinal cord affected the input of sensory information to the right brain. Thus, the differences in the GMV of VC between the two groups of animals may also be attributed to visual compensation. In addition, significant structural differences were found in the Ins and S2 between the two groups of animals. Ins and S2 are both main components of the brain network for acute pain^49^, and patients with NP post-SCI usually show structural atrophy in Ins^50–52^ and S2^50,53^. Considering the NT3-induced alleviation of increased sensitivity to hot pain in this study, and the significant association between the GMV of Ins and WTT in both groups. The brain structural alterations in these areas may be related to pain processing after SCI, indicating that the animals in the LC may suffer from more severe NP compared with those in the NT3 group, and this phenomenon can be further explored in future studies.

Structural differences also existed in Cd, MFG, and IFG between the two groups of animals. Cd plays an important role in the maintenance of trunk and limb posture, as well as the accuracy and speed of directional movement^54^. At the same time, it is part of the basal ganglia, which affects movement by thalamic cortical projection^55^. Rao et al.^56^ investigated the alterations of regional homogeneity (ReHo) in rhesus monkeys post-SCI, and they found that compared with the healthy period, the ReHo in the right Cd of rhesus monkeys increased significantly at 12 weeks after SCI. Therefore, the structural differences in Cd might be due to the compensatory cortical activation following motor impairment in the right lower extremity of the animals. MFG is involved in executive function and emotional regulation^57,58^, whereas IFG is involved in resolving the conflict between motor intention and sensory feedback^59^. Many studies have reported the atrophy of GMV of MFG and IFG in SCI patients compared with the control^14,21,46,60^. Our previous publication has demonstrated that NT3/chitosan treatment can promote the recovery of motor function in animals^31^, and these structural alterations in motion-related brain regions may contribute to this process.

In addition, within 1 year after injury, significant differences occurred in the spatiotemporal patterns of longitudinal brain structural alterations between the animals in LC and NT3 groups. The GMV of the left SPL in NT3 animals showed a significant linear increase with time. A functional overlap existed between SPL and the areas involving motor execution, motor observation, and motor imagination, such as dorsal premotor area, supplementary motor area (SMA), and M1^61^. Therefore, the alterations in GMV of the left SPL in this experiment may be due to the following reasons. After the right hemi-transection of the spinal cord, more intense neuronal activity occurred in the left SPL to maintain the motor function of the right lower extremity^62^. Similar results were reported by Nakanishi et al.^63^, who found that patients with complete SCI showed greater grip strength control over their entire upper extremity compared with healthy controls. Meanwhile, the GMV of bilateral SPL in patients was larger, and the functional connectivity with M1 was stronger. In addition, SPL primarily receives fibers from neighboring S1. The right hemi-transection of spinal cord may result in a decrease in the afferent input to the right S1, thereby affecting the neuronal activity of the right SPL^64^. This phenomenon may have caused the significant increase in GMV alteration of the NT3 group in the right SPL compared with that of the LC, indicating that the ascending sensory conduction pathway in NT3 animals might have been relatively well recovered.

The GMV alterations of left SFG, IFG, and ACC in NT3 animals were significantly higher than those of the LC animals. SFG is involved in various cognitive and motor control tasks^65,66^, whereas IFG is involved in resolving the conflict between motor intention and sensory feedback^59^. Structural atrophy in these regions after SCI has been widely reported^14,21,46^. ACC is involved in the encoding of emotional information on pain and descending pain modulation^49^, and its structural atrophy is common in NP patients post-SCI^20,50^. Asemi et al.^67^ indicated that ACC played a role in motion control through directional or non-directional interaction with the SMA. The differences in the longitudinal brain structural alteration tendencies in these regions between the two groups indicated that NT3-induced regeneration might have a great impact on the motion control and NP post-SCI. The structural alteration rate of ACC in NT3 animals was more stable compared to LC. Considering the wide structural plasticity in brain during rehabilitative treatment after SCI^26,27^ and the protective effect of the microenvironment created by NT3 treatment on the spinal cord structure^30^, it might indicate that NT3 provide some indirect protective effect on the brain structure.

Differences in spatiotemporal patterns of longitudinal spinal cord structural alterations were found between the animals in LC and those in NT3 group within 1 year after injury. SCA atrophy in the epicenter of injury area in NT3 animals was significantly lower than that in LC, which was consistent with the findings of our previous publication^31^. The variability of the alteration rate in the rostral LRW and caudal APW in NT3 animals was also significantly lower than those in LC animals, which indicated that the spinal cord structural changes in NT3 group were relatively stable, thereby reflecting the protective effect of the microenvironment created by NT3 treatment on the spinal cord structure^30^. Notably, compared with the progressive atrophy in animals of the LC after injury, the caudal LRW of the NT3 group first increased significantly and then gradually returned to pre-injury levels. This finding might be due to the fact that the descending motor pathways of rhesus monkeys were located on the left and right sides of the spinal cord. After the right hemi-transection of spinal cord, the motor conduction pathways in animals of the LC were damaged below the injury level, and the caudal LRW was also reduced to a certain extent. However, under the protection of the implanted bioactive materials, the inflammatory responses were inhibited in NT3 animals. Moreover, neuronal death was reduced, and new neurogenesis was enhanced^30^, which may have led to the increase in the left–right width of the spinal cord in the early stage after injury. Then, with the development of new nerves, the ineffective structural connections gradually disappeared, and only a part of completely myelinated axons capable of effective neurotransmission was preserved^31^. This might be the reason for the gradual reduction of the left–right width of the spinal cord in the latter stage after injury.

A significant correlation exists between the GMV alterations and the spinal cord structural alterations in LC and NT3 animals after injury, which may be attributed to the close structural and functional association between the brain and the spinal cord. Brain regions significantly correlated with the spinal cord structural alterations in both groups, including right S1, SFG, Cd, and VC. In this study, no significant alterations in GMV of S1 were detected after injury, which was consistent with the findings of previous publications^18,68,69^. However, in LC, we detected the significant correlation between the alterations in right S1 and rostral SCA and APW. However, in NT3 group, a significant correlation was found between the alterations in right S1 and rostral LRW. This finding may indicate the feasibility of evaluating the structural status of the spinal cord by S1. One of the reasons for the significant correlation between GMV alterations in the right SFG and spinal cord structural alterations may be the fact that SFG holds a complete somatotopical representation of body movements through direct connections with the M1 and spinal cord^70^. Therefore, structural alterations in the spinal cord may affect GMV changes in SFG. Cd and Pu are collectively referred to as the neostriatum, and LGP is referred to as the old striatum. As they are important conduction centers of extrapyramidal system, they cooperate with pyramidal system to complete the management and control of somatic skeletal muscle movement. Therefore, the significant correlation between Cd and spinal cord structural alterations may be due to their structural association through the descending motor pathways^56^.

The specific brain regions in NT3 animals that were significantly correlated with spinal cord structural alterations included right M1 and Th. One of the basic functions of M1 is to control voluntary movement^71^. The right hemi-transection of spinal cord destroys the descending corticospinal tract, which in turn affects the neuronal activity in the contralateral M1. We expected to detect significant alterations in GMV at the left M1, but we did not find any significant difference in GMV at bilateral M1 intra- or inter- groups. According to the review of Nardone et al.^60^, results of studies on gray matter atrophy in M1 post-SCI were relatively divergent. Wrigley et al.^22^ investigated patients with complete thoracic spinal cord injury and found that a significant decrease in GMV could be detected in the M1 of patients with complete SCI. They suggested that the difficulty in detecting significant GMV alterations in M1 in previous VBM studies might be due to the patient’s mixed injury severity, i.e., both complete and incomplete. In the current experiment, we only hemi-transected the spinal cord. Thus, the injury severity was significantly lower than that in the case of complete SCI. This might be one of the reasons why we did not detect a significant alteration. At the same time, we detected a significant correlation between the GMV alterations in the right M1/S1 and M1/SFG and the alterations in rostral LRW and caudal SCA in the NT3 group; however, the peak voxels of these clusters were located at the right S1 and SFG, respectively. As pointed out in the previous discussion, S1 and SFG play an important role in the processing of sensorimotor tasks^18,65^. M1 is a key node in the brain regional network that is responsible for voluntary movement; moreover, it receives and integrates sensory information from different parts of the body to produce appropriate responses^72,73^. Therefore, the clusters that structurally span M1, S1, and SFG may reflect the complex mixing effect of sensorimotor processing post-SCI.

Th is the most important site for converting and integrating sensory afferent information. The input from somatosensory fibers, such as spinothalamic tract, is received at Th; the input is then converted and projected to S1^18^. Although a clear association between Th and spinal cord structural alterations was detected in the NT3 animals, no significant changes in GMV of Th were found after injury. This finding was consistent with the results of previous publications that did not report significant alterations in the Th structure^69,74^ and function^68,74,75^ post-SCI. Chen et al.^18^ suggested that apart from the classical somatosensory pathway, another pathway may exist in the process of sensory-related brain changes after SCI, namely, the cortico-ponto-cerebellar pathway^76^. Sensory function is reorganized after SCI. Although sensory input from the spinothalamic tract to Th is limited, Th may still play an important role in sensory transmission through the cortico-ponto-cerebellar pathway^18^. Therefore, the GMV of Th may not change significantly.

Further investigations on the GMV alterations in overall brain regions showed that for both groups, a significant correlation was preserved in the right S1 and SFG. Therefore, the GMV changes in S1 and SFG may have a certain degree of prediction effect on the spinal cord structural alterations.

This study has some limitations. First, this research was based on a specific injury model (spinal cord hemi-transection and remove model of rhesus monkeys), and the sample size was relatively small, which was different from the more complex and diverse injury scenarios in clinic. Therefore, experimental verifications are needed to determine whether the conclusions drawn in this study can be converted to clinical practice. Second, the voxel size of 0.5 mm^3^ was employed in the study, which was common used in similar research. However, a smaller resolution would lead to more accurate results. And only the alterations of gray matter structure were studied, whereas the white matter could be included in the subsequent studies to reflect the changes in brain structure more comprehensively. Third, the implantation of NT3/chitosan would make it difficult to extract spinal cord automatically at the epicenter of the injury area. However, manual outlining processes ensured the accuracy of spinal cord measurements. Finally, the pathophysiological verification may further reveal the structural alterations in both brain and spinal cord. The combination of MRI and pathophysiological results will help to elucidate the potential biological significance of the extensive MRI characterization in the microenvironment created by the NT3/chitosan-induced regenerative treatment.

## Conclusion

In conclusion, we showed that within 1 year post-SCI, significant differences were found in the effects of NT3-induced regeneration and injury on the brain of animals. This was reflected in the differences in gray matter structure at each time point and in the differences in gray matter structural alteration rate within 1 year after injury. Moreover, differences were found in brain regions related to the integration of motor information and neuropathic pain. Particularly, the decreased GMV of insula was significantly associated with the increased pain sensitivity. Spinal cord structural changes were also influenced by injury and NT3 treatment, and the alterations were significantly associated with the GMV changes in S1, SFG, and other regions. This study may further elucidate the process of structural alterations in the brain after SCI and during its regeneration. Results provide the basis for revealing the unique effects of NT3/chitosan-induced regeneration on the brain structure after SCI.

## Supplementary Information

Supplementary Figures.

## Data Availability

The datasets used and/or analysed during the current study are available from the corresponding author on reasonable request.

## Abbreviations

ACC: Anterior cingulate cortex
APW: Anterior–posterior width
Cd: Caudate nucleus
CST: Corticospinal tract
DARTEL: Diffeomorphic anatomical registration through exponentiated lie algebra
DPABI: Data processing and analysis for brain imaging
FC: Functional connectivity
FWHM: Full width at half maximum
GMV: Gray matter volume
GRF: Gaussian random field
IFG: Inferior frontal gyrus
Ins: Insula
K-S: Kolmogorov Smirnov
L: Left
LC: Lesion control
LGP: Lateral globus pallidus
LRW: Left–right width
M1: Primary motor cortex
MFG: Middle frontal gyrus
MPRAGE: Magnetization-prepared rapid acquisition gradient echo
mVN: Medial vision network
NP: Neuropathic pain
NSC: Neural stem cell
NT3: Neurotrophin-3
Pu: Putamen
R: Right
ReHo: Regional homogeneity
ROI: Regions of interest
S1: Primary somatosensory cortex
S2: Secondary somatosensory cortex
SCA: Spinal cord area
SCI: Spinal cord injury
SFG: Superior frontal gyrus
SMA: Supplementary motor area
SPL: Superior parietal lobule
SPM: Statistical parametric mapping
SST: Study-specific template
TE: Echo time
Th: Thalamus
TI: Inversion time
TR: Repetition time
VBM: Voxel-based morphometry
VC: Visual cortex
WTT: Withdrawal thermal thresholds
