# Supplementary material for: NT3 treatment alters spinal cord injury-induced changes in the gray matter volume of rhesus monkey cortex
Source: Sci Rep. 2022 Apr 8;12:5919. doi: 10.1038/s41598-022-09981-5 (PMC8993853; doi:10.1038/s41598-022-09981-5)
Supplement: Supplementary file 1 — Supplementary Figures. [file 41598_2022_9981_MOESM1_ESM.pdf]

Supporting Information

# NT3 treatment alters spinal cord injury-induced changes in the gray matter volume of rhesus monkey cortex

Shu-Sheng Bao, Can Zhao, Hao-Wei Chen, Ting Feng, Xiao-Jun Guo, Meng Xu, and Jia-Sheng Rao

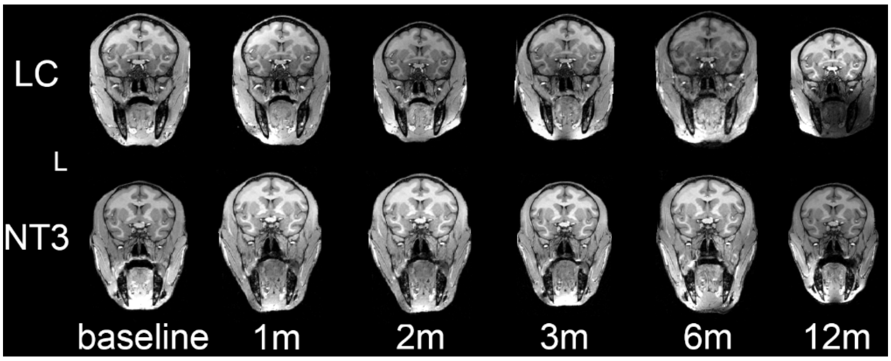


**Fig. S1.** Raw brain images of each time point were displayed for a LC animal and a NT3 animal. L indicated the left side; m represented the month. LC: lesion control; NT3: neurotrophin-3.

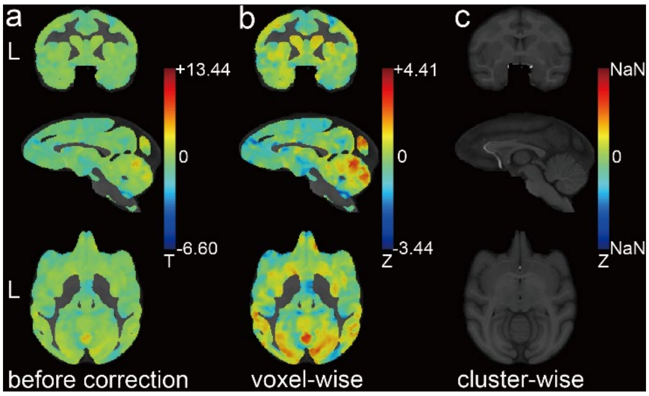


**Fig. S2.** VBM showing the results of the comparisons in global GMV between the LC and NT3 groups at the baseline overlaid on the T1 template image. a) The results before GRF correction. b) The results after voxel-wise correction. c) The results after voxel- and cluster-wise correction. No significant cluster was observed. Two-sample t-test with GRF correction was employed. The voxel level was set to $p<0.005$, and the cluster level was set to $p<0.05$. A positive value in the T or Z-bar indicated that LC > NT3; L indicated the left side.

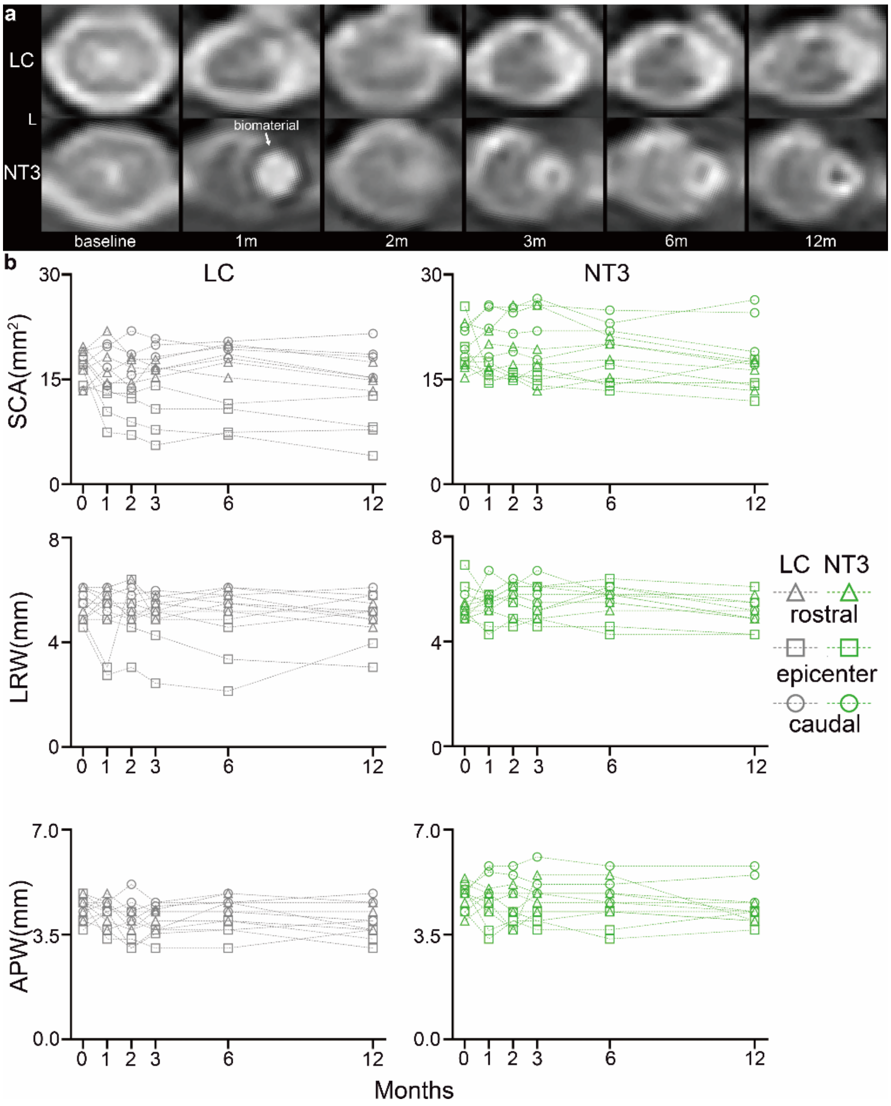


**Fig. S3.** The measurements of spinal cord structural parameters in the LC and NT3 animals. a) Spinal cord images of each time point at the epicenter of the injury area were displayed for a LC animal and a NT3 animal. The white arrow indicated the implanted NT3/chitosan biomaterial. b) Results of longitudinal spinal cord structural alteration tendencies in the two groups. The parameters were obtained at the epicenter, 2 cm rostral, and 2 cm caudal of the injury area. L indicated the left side; m represented the month. LC: lesion control; NT3: neurotrophin-3; SCA: spinal cord area; LRW: left-right width; APW: anterior-posterior width.

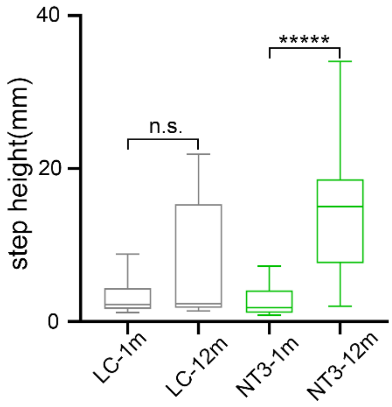


**Fig.S4.** Step height differences at 1 and 12 months after SCI within the LC and NT3 groups (LC: two-sample K-S Z test; NT3: two-sample t-test). *****, $p < 0.00001$; m represented the month; LC: lesion control; NT3: neurotrophin-3.
